# Detection of a *Planktothrix agardhii* Bloom in Portuguese Marine Coastal Waters

**DOI:** 10.3390/toxins9120391

**Published:** 2017-12-03

**Authors:** Catarina Churro, Joana Azevedo, Vitor Vasconcelos, Alexandra Silva

**Affiliations:** 1 Laboratório de Fitoplâncton, Departamento do Mar e Recursos Marinhos, Instituto Português do Mar e da Atmosfera, Rua Alfredo Magalhães Ramalho, 6, 1449-006 Lisboa, Portugal; amsilva@ipma.pt; 2 Centro Interdisciplinar de Investigação Marinha e Ambiental, CIIMAR/CIMAR, Terminal de Cruzeiros do Porto de Leixões, Av. General Norton de Matos, 4450-208 Matosinhos, Portugal; jazevedo@ciimar.up.pt (J.A.); vmvascon@fc.up.pt (V.V.); 3 Departamento de Biologia, Faculdade de Ciências, Universidade do Porto, Rua do Campo Alegre, 4069-007 Porto, Portugal; 4 Centro de Ciências do MAR, CCMAR, Universidade do Algarve, Campus de Gambelas, 8005-139 Faro, Portugal

**Keywords:** *Planktothrix agardhii*, microcystins, *mcy*A, halotolerance, salinity, harmful algal blooms, cyanobacteria

## Abstract

Cyanobacteria blooms are frequent in freshwaters and are responsible for water quality deterioration and human intoxication. Although, not a new phenomenon, concern exists on the increasing persistence, scale, and toxicity of these blooms. There is evidence, in recent years, of the transfer of these toxins from inland to marine waters through freshwater outflow. However, the true impact of these blooms in marine habitats has been overlooked. In the present work, we describe the detection of *Planktothrix agardhii*, which is a common microcystin producer, in the Portuguese marine coastal waters nearby a river outfall in an area used for shellfish harvesting and recreational activities. *P. agardhii* was first observed in November of 2016 in seawater samples that are in the scope of the national shellfish monitoring system. This occurrence was followed closely between November and December of 2016 by a weekly sampling of mussels and water from the sea pier and adjacent river mouth with salinity ranging from 35 to 3. High cell densities were found in the water from both sea pier and river outfall, reaching concentrations of 4,960,608 cells·L^−1^ and 6810.3 × 10^6^ cells·L^−1^ respectively. Cultures were also established with success from the environment and microplate salinity growth assays showed that the isolates grew at salinity 10. HPLC-PDA analysis of total microcystin content in mussel tissue, water biomass, and *P. agardhii* cultures did not retrieve a positive result. In addition, microcystin related genes were not detected in the water nor cultures. So, the *P. agardhii* present in the environment was probably a non-toxic strain. This is, to our knowledge, the first report on a *P. agardhii* bloom reaching the sea and points to the relevance to also monitoring freshwater harmful phytoplankton and related toxins in seafood harvesting and recreational coastal areas, particularly under the influence of river plumes.

## 1. Introduction

Toxic cyanobacterial blooms have long been a recognized threat to freshwater ecosystems and human health, and their negative impacts have been reported and reviewed extensively [1,2,3,4,5,6,7]. These toxic proliferations are still a major concern in freshwater lakes and reservoirs and their complexity and unpredictable nature continue to challenge researchers and monitoring authorities [8]. The occurrence, distribution, magnitude, and persistence of these blooms are perceived to be increasing globally over the last years [8,9,10,11,12,13] and have been related to increasing human activities and global warming [9,10,11,12,13,14]. However, cyanobacterial proliferations are intricate phenomena and their occurrence is the result of the juncture of several factors, related but not exclusive to, increasing/reduction of nutrient inputs, increasing river barriers, increased water stratification periods, changes in the hydrological cycle, increasing global temperatures, and CO_2_ [9,10,11,12,13,14,15,16]. Whether or not this is connected to climate change massive cyanobacterial blooms are no longer seasonal, persisting during all year, and also, not restrict to lentic water systems where rivers and estuaries have also been affected [8,9,17,18]. The reports of freshwater cyanobacterial blooms reaching the sea are increasing and have been most probably underestimated [19,20,21,22]. Furthermore, the effect of these blooms in the marine ecosystem and the impact in human and wildlife are largely unknown [19,20,21,22]. The most commonly found freshwater toxins—microcystins—are produced by several bloom-forming cyanobacteria species, such as *Microcystis*, *Planktothrix*, *Anabaena*, *Anabaenopsis*, and *Aphanizomenon*. These genera—*Microcystis* and *Planktothrix*—are globally distributed and form blooms in lakes and reservoirs all over the world. In freshwater, the main concern from microcystin intoxication is through ingestion or dialysis of/with contaminate drinking water, recreational contact and animal poisoning [6,23]. These hepatotoxins are known to be chemically stable in both fresh and marine water, to persist in sediments, to accumulate in both freshwater and marine filter-feeders and to transfer up the food chain [19,20,21,24,25,26]. In a recent review by Preece et al. [22] the authors describe freshwater blooms occurrences in estuarine and coastal waters in North and South America, Europe, Africa, Australia, Turkey, and Japan. In some of the reports, microcystins were detected in the water and were accumulating in marine shellfish with animal deaths implicated [22]. Some countries, like Australia and USA, have recently defined microcystins guidelines for fish, prawns and molluscs to apply in some states [26,27]. In general, however, governments, food standards regulations and legal guidelines for seafood safety do not yet include freshwater phytoplankton related toxins [23,27]. In the same way, the Portuguese legislation only includes the obligation of monitoring marine phytoplankton related toxins in seafood through the Law Decree n.º 293/98 (1998) [28] transposed from the European Directive n.º 91/492/CEE (1991) [29]. The Portuguese legislation concerning cyanobacteria and microcystins is only related to drinking water safety (Law Decree n.º 306/2007 [30] transposed from the European Drinking Water Directive [31]) and the legislation concerning the quality of bathing water [32,33], that was transposed from the European Bathing Water Directive [34], poorly addresses this question. In the light of what appears to be an additional and increasing concern, it is important to report freshwater cyanobacteria blooms in coastal waters and to access recreational and seafood safety. In the present work, we describe the occurrence of a freshwater cyanobacterium bloom in marine water and land-sea interface at a shellfish harvesting and bathing area located on the W coast of Portugal. We went further testing cell concentration and microcystin content in the water and shellfish, cyanobacteria strain viability in a marine environment with contrasting salinities and the presence of microscystin related genes to evaluate potential toxic impact in the ecosystem.

## 2. Results

In November of 2016, during the regular monitoring program for the screening of harmful marine phytoplankton, a high concentration of the filamentous cyanobacteria *Planktotrix agardhii* (4,960,608 cells·L^−1^) was observed in a marine sample at the northwest Portuguese coast (geographical coordinates: 39°21′00.4″ N 9°22′19.4″ W). The area is a shellfish harvesting site and a recreational sea beach used for bathing and surfing.

**Figure 1:**
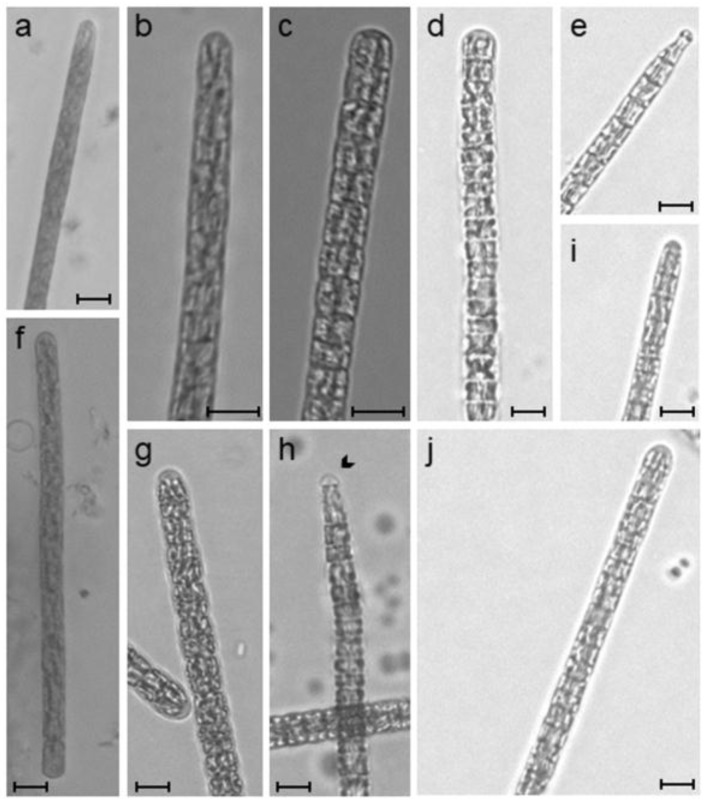
Light microscopy photographs of *Planktothrix agardhii* strains observed in this study. (**a**,**f**)—*P. agardhii* filaments observed in marine water from the sea pier; (**b**,**c**)—*P. agardhii* filaments observed in freshwater from S. Domingos reservoir; (**d**,**e**)—*P. agardhii* strain IPMA2; (**g**,**h**)—*P. agardhii* strain IPMA5; (**i**,**j**)—*P. agardhii* strain IPMA3. The arrow indicates the calyptra. Scale bar 5 µm.

The observed cyanobacteria are from the group Oscillatoriales with no heterocytes nor akinetes, the filaments were blue-green, solitary and without sheaths. The filaments were continually attenuated at one extremity and straight at the other, the cells were 3.54 ± 0.365 µm wide and 2.65 ± 0.467 µm long with no constrictions at cross-cell walls, the apical cell was narrowed at one side of the filament with calyptra and rounded at the other side (Figure 1a,f). These morphological characteristics were in congruence with the description of the cyanobacterium *Planktothrix agardhii* a typically freshwater organism known to produce the hepatotoxins Microcystins. 

Following this observation, the site was monitored closely. Samples were collected at three sites: at the sea pier (salinities from 35 to 37), beach (salinities from 7 to 13) and river outfall (salinities from 3 to 5). One sample was taken in the freshwater reservoir located inland at the same water line (salinity 0). 

**Figure 2:**
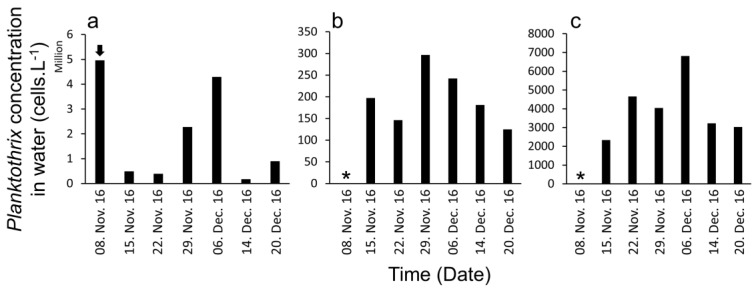
*P. agardhii* cell concentrations in water samples. (**a**)—Sampling point 1: monitoring station of the Portuguese National Shellfish Monitoring System at the sea pier; (**b**)—Sampling point 2: River runoff at the beach between the sea and the river; (**c**)—Sampling point 3: river outfall near the beach. The arrow indicates the first observed occurrence; (*)—No sampling.

Cell concentrations in water were high at all the locations and present during all sampling period but with very different orders of magnitude, decreasing from the river outfall to the sea (Figure 2). At the sea pier, cell concentrations showed high oscillations weekly (Figure 2a) and were between 4,960,608 cells·L^−1^ and 174,244 cells·L^−1^ (Figure 2a). At the beach the cell concentrations were higher than the sea pier, ranging from 296.229 × 10^6^ cells·L^−1^ to 124.571 × 10^6^ cells·L^−1^ (Figure 2b). At the river outfall, *P. agardhii* concentrations were the highest recorded, between 6810.3 × 10^6^ cells·L^−1^ and 2328 × 10^6^ cells·L^−1^ (Figure 2c) with orders of magnitude similar to the freshwater reservoir (8935.7 × 10^6^ cells·L^−1^).

Filaments were isolated from the water to freshwater Z8 culture media. Cultures were established with success indicating that *P. agardhii* is preferably a freshwater species. Cultures were also obtained from all the locations including the sea pier, indicating that the cells were viable at the sea water with a salinity of 35. The list of *P. agardhii* cultures established and used in this study is displayed in Table 1. 

Figure 1 represents the specimens observed from both environmental samples and clonal cultures. The morphology of the *P. agardhii* strains maintained in culture was as described previously for the observed wild specimens. 

**Figure 3:**
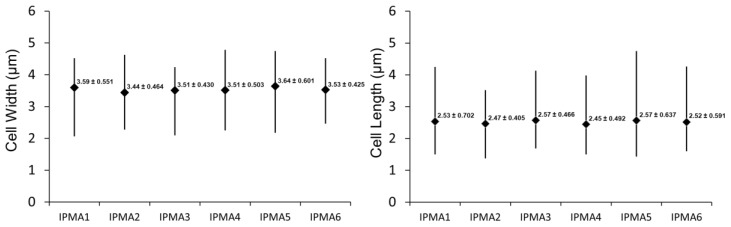
Morphometry of *Planktothrix agardhii* strains isolated in this study. Narrow lines indicate the range of the measurements; (♦)—indicates the average with the value and the standard deviation.

The morphometry of the strains was between 3.44 ± 0.464 and 3.64 ± 0.601 for cell width and between 2.45 ± 0.492 and 2.57 ± 0.466 for the cell length (Figure 3). No significant differences were obtained between strains for cell width (ANOVA; F_5, 600_ = 1.682; *p* > 0.05) and cell length (ANOVA; F_5, 600_ = 0.845; *p* > 0.05).

**Figure 4:**
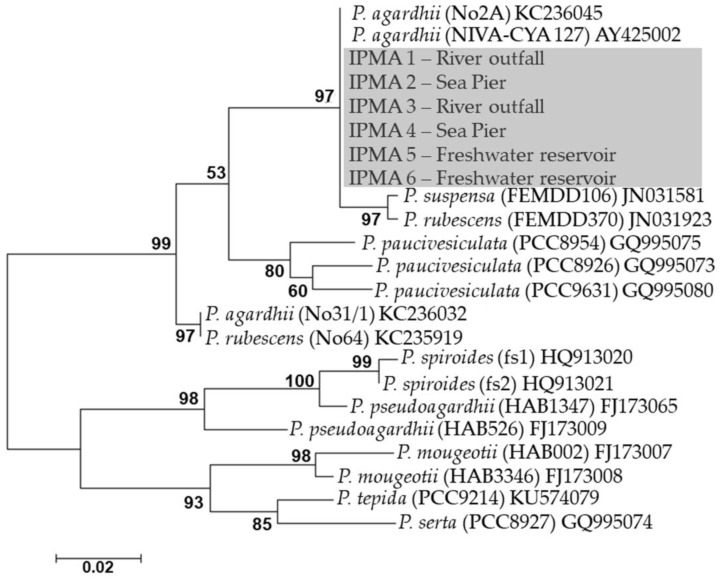
Phylogenetic tree of *P. agardhii* strains and closely related *Planktothrix* retrieved from GenBank, inferred by using the Maximum Likelihood method based on the Kimura 2-parameter model of the *rpo*C1 gene sequences. The percentage bootstrap values of 1000 replicates are given at each node. GenBank accession numbers are indicated after the species designation. The tree is unrooted and drawn to scale, with branch lengths measured in the number of substitutions per site. The analysis involved 23 nucleotide representative sequences. There were a total of 452 positions in the final dataset. The grey box indicates the origin and strain number of the cultures obtained in this study. Accession numbers: IPMA1—MG452723; IPMA2—MG452724; IPMA3—MG452725; IPMA4—MG452726; IPMA5—MG452727; IPMA6—MG452728.

Since morphology of *Planktothrix* is very similar between species the molecular analysis of the *rpo*C1 gene was performed and confirmed the identification of the cultures also as *P. agardhii* by BLAST search. The phylogenetic analysis showed that our strains form a well-supported monophyletic clade in one of the unresolved groups of the *P. agardhii*/*P. rubescens* complex (Figure 4). *P. agardhii* and *P. rubescens* are difficult to distinguish molecularly, differing morphologically by its color, one is green and the other is red [9,35].

**Figure 5:**
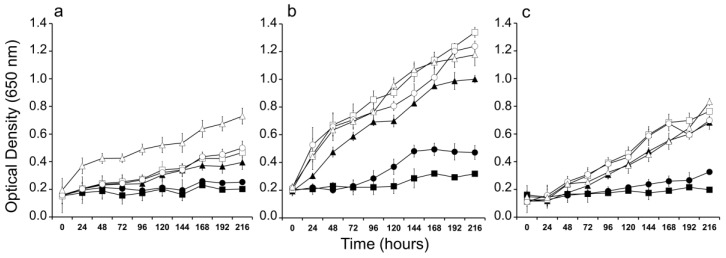
Growth curves of the *Planktothrix agardhii* strains exposed to several salinities. (**a**) *P. agardhii* IPMA2 collected at the sea pier; (**b**) *P. agardhii* IPMA3 collected at the river outfall; (**c**) *P. agardhii* IPMA5 collected at the freshwater reservoir. Range of salinities tested: (∆) 0; (○) 2.5; (□) 5; (▲) 10; (●) 20; (■) 30.

*P. agardhii* growth, exposed to a range of salinities was evaluated using optical density measurements over a 216 h period (Figure 5). The growth pattern for all the strains tested (IPMA2; 3 and 5) was similar regardless the origin of the strain (sea pier, sampling point 1—Figure 5a; river outfall, sampling point 3—Figure 5b and the freshwater reservoir, sampling point 4—Figure 5c). The strains grew well in salinities between 0 and 10. Growth inhibition was visible at salinities 20 and 30 for all the strains tested (Figure 5).

No microcystins were detected in the tissues from mussels and the biomass from water and cultured strains. Furthermore, the microcystin related gene (*mcy*A) was also not detected in water nor cultures, indicating that no toxic strains were present in the environment. 

## 3. Discussion

The possible human health risk of freshwater blooms in coastal environments and consequent contamination of marine shellfish has recently received attention with the death of a large number of sea otters with liver failure in Monterey Bay, California after ingestion of microcystin contaminated shellfish [36]. Furthermore, recreational exposure incidents of acute illness in humans have been reported for the La Plata estuary [7]. In Portugal, the reports of freshwater cyanobacterial blooms in estuaries were related with the cyanobacterium *Microcystis*. Until now, were reported in Minho estuary where a *Microcystis* bloom was transported through the river [37] and in Guadiana estuary where *Microcystis* blooms often develop and accumulate in the upper part of the estuary [38,39]. Microcystins have never been detected in the lower part of the estuaries, marine water nor marine shellfish [37,38]. In this work, we found that the bloom-forming cyanobacterium *Planktothrix agardhii* is reaching sea waters at high cell concentrations and that the surrounding area at the land-sea interface is heavily loaded with *P. agardhii* cells. The cell densities found in the water, from the river outfall, beach and sea were above the cell limit of 2 × 10^6^ cells·L^−1^ recommended by WHO, from which microcystin concentration in water can exceed the WHO guideline of 1 µg·L^−1^ of safe daily ingestion by an oral root [3]. Gible and colleagues [21] found that mussels fed with 2 × 10^9^ cyanobacterial cells·L^−1^, with an average of 5.6 µg·L^−1^ total microcystins for 24 h could accumulate 4 to 6 ng of microcystins per g of tissue. Mulvenna and colleagues [40] calculated derived guideline values for microcystins in seafood of 51 µg of microcystins per Kg. Given the cell concentrations obtained, our results indicate that this area is vulnerable to shellfish contamination by freshwater toxins if present, despite no microcystins nor microcystin related genes were observed. Concerning WHO recommendations for bathing waters, the cell concentrations in our study at the sea pier, were below 2 × 10^7^ cells·L^−1^ guideline that represents a low risk of adverse effects from cyanobacteria exposure [3]. However, at the beach and in the river outfall cell concentrations were above the 1 × 10^8^ cells·L^−1^ guideline which constitutes a high risk according to WHO [3]. The studied site is not only a shellfish harvesting area but also a recreational sea beach used for bathing and known as a good site for surfing after what is called “Praia dos Supertubos”. The recreational use of this beach goes beyond the seasonality of the summer and is used by surfers, surf schools, and surf competitions during all year around.

*P. agardhii* is considered a freshwater cyanobacterium, but, is a resilient and persistent species known to tolerate a wide range of temperatures and light intensities, meaning that has been found in a wide range of environments prevailing all year. It has been found blooming under ice-covered lakes in Poland, mixed in the water column in eutrophic lakes or to form metalimnetic blooms in more oligotrophic and/or water stratification conditions [41,42,43,44,45]. In this study, the possible source of the cells is the freshwater reservoir located 5 km upstream as it contains elevated cell densities of *P. agardhii*. Most probably the cells found in the sea belong to a population continually renewed by the input from the river. The salinity tolerance tests showed that all strains grown in salinities of 10, including the freshwaters reservoir strain. These results point out that *P. agardhii* may have the capability of growing in the river outfall, where salinities vary from 3 to 5. Furthermore, the ability of the freshwater strain also to develop in moderate salinities of 10 implicates a continuous flow of *P. agardhii* to the sea water, as the species proliferate through transitional waters. This resilience to salinity has been reported by several authors who verified a salinity growth range between 0 and 7.8 [35,46,47]. Blooms of *P. agardhii* have been reported for low salinity waters, such as, the Bolmon lagoon that has salinities from 5 to 10, Olivier pond with salinities between 2.8 and 3.9 and Albufera de Valencia with salinities between 1 and 3 [47,48,49]. In addition, a massive bloom of the closely related *P. rubescens* in Lake Occhito was reported to reach the sea contaminating mussel farms in Italy [50]. In the light of our and the aforementioned results, it is important to understand the halotolerance of this species and differences between toxic and non-toxic strains to understand their ability to colonize more saline environments.

The Portuguese legislation has the obligation to monitoring cyanobacteria and Microcystins only related with drinking water safety for freshwater reservoirs and the legislation concerning seafood safety does not include freshwater toxins [28,30]. Given the results obtained in this study, the *P. agardhii* present in the environment was probably a non-toxic strain, but the concentrations were alarming for coastal areas especially from a species that is very often reported as toxic. Toxic and non-strains of cyanobacteria are morphologically indistinguishable and toxin analysis must be mandatory. We believe that cyanotoxin occurrence and concentration in marine waters and shellfish are pertinent to be monitor routinely as these blooms are unpredictable. Also, according to Portuguese legislation for recreational waters quality [32,33], sampling should only be carried out when a proliferation is observed, with no guideline for concentration levels. In the case of our study, per example, visual inspection of the water is not adequate since, usually, *P. agardhii* blooms do not form aggregates, scums or foams at the surface being generally dispersed through to the water column in high cell densities [3].

This work reinforces the need for a regular surveillance plan to monitor the presence of cells and toxins in marine water for recreational and environmental purposes as well as for seafood safety. It also demonstrates the relevance of monitoring transitional waters with a sequence of stations upstream enough to act as early-warning stations of blooms reaching coastal areas. 

## 4. Materials and Methods 

### 4.1. Location and Sampling

**Figure 6:**
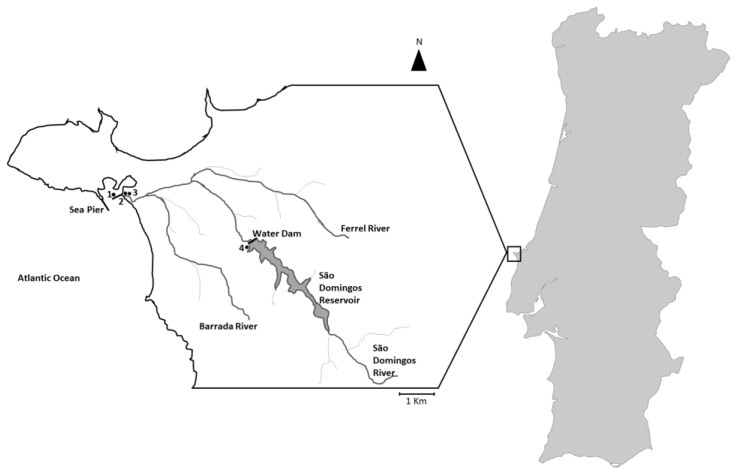
Schematic representation at scale of the sampling site at the northwest of the Portuguese coast. (●1)—Monitoring station of the Portuguese National Shellfish Monitoring System at the sea pier (39°21′00.4″ N 9°22′19.4″ W), (●2)—Sampling point 2, river run-off at the beach between the sea and the river (39°21′03.0″ N 9°22′04.7″ W), (●3)—Sampling point 3 at the river outfall near the beach (39°21′04.6″ N 9°21′58.6″ W), (●4)—Sampling point 4 near the water dam discharged at the freshwater reservoir located 5 km upstream of the river S. Domingos (39°19′58.1″ N 9°19′07.5″ W), (**−**)—Sea Piers and water dam, grey lines represent water lines.

Cyanobacteria were first observed in Marine waters from the northwest Portuguese coast in the beginning of November of 2016. The observation site is part of the Portuguese National Shellfish Monitoring System that is sampled every week for the screening of harmful marine phytoplankton and related toxins [51]. The region surrounding the sampling site is represented in Figure 6. It is near the outfall of the river S. Domingos that is characterized by a water dam 5 km upstream and the tributary of two other rivers (Figure 6). Between November and December of 2016, the samples were taken every week at three different locations at the site (Figure 6). At sampling point 1 (sea pier) both water and mussels were sampled. At sampling points 2 at the beach in the river run-off and 3 river out-fall, only water was sampled (Figure 6). The freshwater reservoir locates 5 km upstream in the river was sampled once to investigate the presence of cyanobacteria (Figure 6). All the water samples were taken at the surface in low tide, transported to the laboratory in refrigerated conditions and the salinity measured with a hand-held visual refractometer, Index Instruments LTD. A part was filtered for toxin and molecular analysis, part was preserved for cell counting and another part was kept fresh for filament isolation and culture establishment.

### 4.2. Filament Isolation and Culture Establishment

Cyanobacteria filaments were isolated with a micropipette under the inverted microscope Leica^®^ DMi8 from water fresh samples concentrated with a 10 µm net. Cultures were established by transferring the isolated filaments into Z8 culture medium [52] at salinities 35 and 0. Successful cultures were maintained at 19 ± 1 °C with a light intensity of 5 μmol·photons·m^−2^·s^−1^ and a 12:12 h light:dark cycle.

### 4.3. Cyanobacteria Identification and Quantification

For the quantification of cyanobacteria, the samples were preserved with neutralized Lugol’s iodine solution, settled down in sedimentation chambers (25 mL) and counted using an inverted microscope Leica^®^ DMi8 at 400× magnification following the procedure of the Utermöhl technique described in the European Standard EN15204 [53]. The number of cells in oscillatoriales cyanobacteria was calculated by dividing the measured filament length by the mean cell length.

For the morphological characterization, the cyanobacteria were analyzed using a Leica^®^ DMi8 inverted microscope at 1000× magnification. The morphological characters evaluated were: cell dimensions (length and width), filament color and shape, constrictions at the cell wall, presence of sheath, shape of apical cell, presence/absence of calyptra and necredia [35,46,54,55]. Photographs were taken with a Leica^®^ DFC550 digital camera. The cell measurements were performed using Leica^®^Lasx software. At least 50 measurements were done for calculating the size of the cells and filaments. Differences in cell dimensions (length and width) were tested with a one-way Analysis of Variance (ANOVA) for *p* < 0.05. 

For the molecular identification, the DNA was extracted from the cultures using the DNeasy Plant Mini Kit, Quiagen^®^. The total DNA concentration was quantified using the Qubit™ Fluorometric Quantitation, Thermo Fisher Scientific^®^. A DNA fragment of 608 bp within the *rpo*C1 gene was amplified with the primers RPOF (5′-TGGTCAAGTGGTTGGAGA-3′) and RPOR (5′-GCCGTAAATCGGGAGGAA-3′) [56]. The amplifications were performed in a reaction mixture containing DreamTaq PCR Master Mix (ThermoFisher Scientific^®^) with 1 U of Taq DNA polymerase using a T100™ Thermal Cycler (BioRad) programmed with a PCR cycle consisting of an initial denaturation step at 94 °C for 3 min, followed by 40 cycles of 20 s at 94 °C, 30 s at 55 °C and 20 s at 72 °C and a final extension step of 5 min at 72 °C. The amplified fragments were visualized under UV light after electrophoretic analysis performed in 1% *w/v* agarose gel with GreenSafe Premium™ DNA staining (NZYTech^®^), at 80 V in 0.5× Tris-borate EDTA (TBE) buffer for 45 min. The amplified PCR products were purified and sequenced by Sanger sequencing in the commercial platform GATC Biotech^®^. 

Sequences of the 608 bp fragment of the *rpo*C1 gene were analyzed with the Basic Local Alignment Search Tool (BLAST™) to access similarity and identity of the sequences with the sequences in the database. A total of 189 sequences of the partial *rpo*C1 gene from *Planktothrix* species were retrieved from the database. The nucleotide alignment was performed with MUSCLE [57] using MEGA7 [58]. A total of 23 sequences were chosen as representatives of each biological nucleotide sequence. A phylogenetic tree was constructed using the Maximum Likelihood method based on the Kimura 2-parameter model [59] using MEGA7 [58]. There were a total of 452 positions matrix in the final dataset and all positions containing gaps and missing data were eliminated. Node support was estimated using 1000 bootstrap replicates. The sequences obtained in this study were deposit in the GenBank^®^ genetic sequence database [60]. Accession numbers: IPMA1—MG452723; IPMA2—MG452724; IPMA3—MG452725; IPMA4—MG452726; IPMA5—MG452727; IPMA6—MG452728.

### 4.4. Toxin Analysis

Microcystins were extracted from (1) 15 g of mashed mussel tissue; (2) the filters (GF/C, microfibre filters, Whatman^®^) with the biomass of 2 L of water and (3) lyophilized *P. agardhii* cultures biomass (100 mg dry weight) with 50% methanol for 2 h under magnetic stirring. The extracts were sonicated (ice bath, 60 Hz, 1 min pulses) with an ultrasonic probe, centrifuged (4495 *g*, 4 °C, 5 min) and the pellets were re-extracted overnight by the same procedure. Supernatants of the two extractions were combined and subjected to rotary evaporation at 35 °C to eliminate methanol. The resulting aqueous extracts were cleaned-up by solid phase extraction on SPE cartridges (Sep-Pak C18, Phenonenex) previously activated with 20 mL of ethanol and equilibrated with 20 mL of distilled water. The microcystin containing fraction was eluted with methanol at 80% (*v/v*) and the methanolic fraction evaporated. The resulting solution was filtered through 0.45 µm syringe filters and analyzed by HPLC-PAD. Microcystins were identified by their characteristic absorption maximum at 238 nm and quantified using commercially available MC-LR standard (Alexis^®^ Biochemicals). The samples were injected in an HPLC system from Waters^®^ Alliance e2695 coupled with a PDA 2998 equipped with on a Merck^®^ Lichrospher RP-18 endcapped column (250 mm × 4.6 mm i.d., 5 µm) equipped with a guard column (4 × 4 mm, 5 µm) both kept at 45 °C. The PDA range was 210–400 nm with a fixed wavelength of 238 nm. The linear gradient elution consisted of (A) methanol + 0.1% trifluoroacetic acid and (B) H_2_O + 0.1% TFA (55% A and 45% at 0 min, 65% A and 35% B at 5 min, 80% A and 20% B at 10 min, 100% A at 15 min, 55% A and 45% B at 15.1 and 20 min) with a flow rate of 0.9 mL·min^−1^. The injected volume was 20 µL. The system was calibrated by using a set of 7 dilutions of MC-LR standard (0.5 to 20 μg·mL^−1^) in methanol 50%. Each vial was injected in duplicate and every HPLC run series of ten samples was constituted with a blank and two different standard concentrations. Empower 2™ Chromatography Data Software was used for calculation and reporting peak information. The detection and quantification limits of MC-LR that can be detected in water and cyanobacteria biomass are 0.3 μg·mL^−1^ and 0.5 μg·mL^−1^, based on a signal-to-noise ratio of 3 and 10. All HPLC solvents were filtered (Pall^®^ GH Polypro, 47 mm, 0.2 μm) and degassed by ultrasound bath.

DNA from the cultures and water filtered biomass (with the filtrate of 1 L of water samples) was extracted as mentioned above. A PCR reaction was performed to check for the presence of a fragment of the *mcy*A gene related with microcystin production. For the reaction, the primers MAPF (5′-CTAATGGCCGATTGGAAGAA-3′) and MAPR (5′-CAGACTATCCCGTTCCGTTG-3′) [61] were used in a mixture containing DreamTaq PCR Master Mix (ThermoFisher Scientific^®^) with 1 U of Taq DNA polymerase using a T100™ Thermal Cycler (BioRad) with a thermocycling profile consisting of an initial denaturation step at 94 °C for 3 min, followed by 35 cycles of 20 s at 94 °C, 20 s at 60 °C, and 20 s at 72 °C and a final extension step of 5 min at 72 °C. DNA from the *P. agardhii* strain CCALA159, that is a microcystin producer [62], was used has a positive control for the amplification. The PCR reactions were checked for fragment amplification under UV light after electrophoretic analysis performed in 1% *w/v* agarose gel with GreenSafe Premium™ DNA staining (NZYTech^®^), at 80 V in 0.5× Tris-borate EDTA (TBE) buffer for 45 min.

### 4.5. Salinity Tolerance Experiments

A 96-well microplate bioassay was used to evaluate *P. agardhii* growth in a salinity gradient (30 to 0). Culture Z8 media was prepared using natural seawater. Aliquots (100 μL) of exponential growing stock cultures were added to microplate wells previously filled with Z8. The total assay volume in each well was 200 μL. Three replicates were used for each experimental condition and 7 for the control condition (salinity 0). The plates were sealed with Parafilm™ to reduce evaporation. Cuts in the Parafilm™ were made to allow gas exchange and avoid condensation. Sealed plates were placed in the culture chamber under the same light and temperature conditions as described for culture maintenance. Optical densities of each well were measured daily for 9 days at 655 nm using a microplate absorbance reader BioRad 680XR. Mean values and the coefficient of variation (standard deviation/mean) of optical density measurements from replicates were calculated and used to estimate culture growth over a 216-h period. Some of the strains maintained in culture (IPMA1; 4 and 6) formed clumps, so, it were not tested for salinity tolerance.

## Figures and Tables

**Table 1:** List of *Planktothrix agardhii* cultures established. River outfall corresponds to the sampling point 3, the sea pier corresponds to sampling point 1 and S. Domingos reservoir is the freshwater lake at sampling point 4.

Strain Number	Origin	Date of Isolation
IPMA1	River outfall	29 November 2016
IPMA2	Sea Pier	8 November 2016
IPMA3	River outfall	29 November 2016
IPMA4	Sea Pier	8 November 2016
IPMA5	S. Domingos reservoir	15 November 2016
IPMA6	S. Domingos reservoir	15 November 2016
